# Surgical Wards of St. Joseph’s Hospital, from May 1st to July 18th

**Published:** 1851-08

**Authors:** R. Durney

**Affiliations:** Resident Physician


*Surgical Wards of St. Joseph's Hospital, from May 1st to July 18th.—Service of* Dr. J. H. B. McClellan. Reported by R. Durney, M. D., Resident Physician.

| Disease. | Cured. | Relieved. | Died. | Removed. | Remaining. | Total |
|---|---|---|---|---|---|---|
| Abscess, mammary - | 1 | 0 | 0 | 0 | 0 | 1 |
| Anchylosis of ankle-joint - | 0 | 1 | 0 | 0 | 0 | 1 |
| " knee " - | 0 | 1 | 0 | 0 | 0 | 1 |
| Artificial anus - - | 0 | 0 | 1 | 0 | 0 | 1 |
| Burns - - - - | 5 | 0 | 0 | 3 | 0 | 8 |
| Caries of phalanges - | 0 | 0 | 0 | 0 | 1 | 1 |
| " keee-joint - | 0 | 0 | 0 | 1 | 0 | 1 |
| " metacarpal bones | 0 | 0 | 0 | 1 | 0 | 1 |
| " metatarsal " | 1 | 0 | 0 | 0 | 0 | 1 |
| Contusion of hip and thigh | 0 | 0 | 0 | 0 | 2 | 2 |
| Conjunctivitis - - | 1 | 0 | 0 | 0 | 0 | 1 |
| Fractures, simple, 13, viz.: | | | | | | |
| Arm - - - - | 1 | 0 | 0 | 0 | 0 | 1 |
| Clavicle - - - | 1 | 0 | 0 | 1 | 2 | 4 |
| Crest of ileum - - | 1 | 0 | 0 | 0 | 0 | 1 |
| Condyle of humerus - | 1 | 0 | 0 | 0 | 0 | 1 |
| Neck " " - - | 0 | 0 | 0 | 1 | 0 | 1 |
| Leg - - - - | 0 | 0 | 0 | 0 | 1 | 1 |
| Nasal bones - - | 1 | 0 | 0 | 0 | 0 | 1 |
| Forearm - - - | 1 | 0 | 0 | 0 | 0 | 1 |
| Femur - - - - | 1 | 0 | 0 | 0 | 0 | 1 |
| Spine - - - | 0 | 0 | 0 | 0 | 1 | 1 |
| Fractures, compound, 4, viz.: | | | | | | |
| Foot - - - - | 0 | 0 | 0 | 0 | 1 | 1 |
| Leg - - - - | 0 | 0 | 1 | 0 | 1 | 2 |
| Comp. comminuted of forearm | 0 | 0 | 0 | 0 | 1 | 1 |
| Fracture, ununited, of leg | 1 | 0 | 0 | 0 | 0 | 1 |
| Fistula in ano - - - | 4 | 0 | 0 | 0 | 0 | 4 |
| " in perineo - - | 0 | 0 | 0 | 1 | 0 | 0 |
| Gonorrhœa - - - | 3 | 0 | 0 | 0 | 0 | 3 |
| Hydrops articuli of knee - | 0 | 1 | 0 | 0 | 0 | 1 |
| Paralysis of bladder - | 1 | 0 | 0 | 0 | 0 | 1 |
| Paronychia - - - | 2 | 0 | 0 | 1 | 0 | 3 |
| Scirrhus testis - - - | 1 | 0 | 0 | 0 | 0 | 1 |
| Sprains - - - - | 2 | 0 | 0 | 0 | 0 | 2 |
| Ulcers - - - - | 1 | 0 | 0 | 0 | 0 | 1 |
| Wounds, simple: | | | | | | |
| Radial artery - - | 1 | 0 | 0 | 0 | 0 | 1 |
| Palmar arch - - | 0 | 0 | 0 | 0 | 1 | 1 |
| Scalp, lacerated - - | 2 | 0 | 0 | 0 | 2 | 4 |
| Foot, " - - | 0 | 0 | 0 | 0 | 2 | 2 |
| Hand, " - - | 1 | 0 | 0 | 0 | 0 | 1 |
| Eye - - - - | 1 | 0 | 0 | 0 | 1 | 2 |
| | 34 | 3 | 2 | 9 | 16 | 64 |

In two of the cases of compound fracture, amputation was found necessary. In one of these—that of a compound fracture of one leg with comminuted fracture of the other limb, from a railroad accident—it was performed on the sixteenth day after admission, in consequence of mortification having taken place. The patient died on the twenty-eighth day with sloughing of the stump. In the other case—a compound and comminuted fracture of the forearm, in which the hand and both bones were completely crushed by being caught in the machinery of a cotton factory—it was performed immediately after reaction, and the patient is now doing well.

The case of ununited fracture of both bones of the leg, was cured simply by the support of splints and bandages steadily continued for a long time. This treatment was adopted at the previous term by Dr. Horner, and was continued in this with complete success.

The case of artificial anus was the result of an operation for strangulated hernia, performed in the city some months previous. The parts around the orifice, which was of considerable size, were very much thickened and indurated; and every effort made to accomplish a cure without resorting to an operation, was without the slightest effect. Fæces were constantly discharging through the wound, and could not be retained or suppressed by any bandage or appliance whatever. The skin for some distance around the orifice became excoriated and tender, and the patient rapidly declining in health, it was determined to perform Dr. Physick's operation, with the hope of affording relief. This operation was performed by dilating the wound or external orifice, so as to apply a ligature to the deeply-seated gut. Owing to the induration of the tissues, and the alteration from the normal condition of the parts, this required considerable care, notwithstanding which the epigastric artery was divided. It was, however, promptly and with little difficulty secured, with very little loss of blood. A ligature was then applied, attached to a curved needle by means of Physick's forceps, through the septum, dividing the double gun-barrel-like orifices of the bowel. The case did not progress favorably after the operation. For about twenty-four hours the patient was apparently doing well, but she gradually became weaker and weaker, and finally exhibiting the characteristic symptoms of peritonitis, died on the sixth day after the operation. On a post mortem examination, the ligature was found in the proper situation, about a quarter to half an inch from the external margin of the septum, and adhesion had already taken place between its layers. The bowels were found to be inflamed some distance both above and below the seat of the operation, with extensive adhesions between their folds.

